# Dengue disease status in Chennai (2006-2008): A retrospective analysis

**Published:** 2011-03

**Authors:** P. Gunasekaran, K. Kaveri, S. Mohana, Kavita Arunagiri, B.V. Suresh Babu, P. Padma Priya, R. Kiruba, V. Senthil Kumar, A. Khaleefathullah Sheriff

**Affiliations:** *Department of Virology, King Institute of Preventive Medicine, Chennai, India*

**Keywords:** Dengue-arboviral infection, dengue haemorrhagic fever (DHF), dengue shock syndrome (DSS)

## Abstract

**Background & objectives:**

Dengue is one of the most important Arboviral diseases in man with outbreaks in Southeast Asia and India. We report a retrospective analysis of the dengue positivity in the referred samples for three years period (2006 to 2008) at the Department of Virology, King Institute of Preventive Medicine, Chennai, Tamil Nadu, India.

**Methods:**

Serum samples from 1593 suspected cases (968 male and 625 female) were obtained. Of the 1593 cases screened, 1204 (75.5%) were paediatric cases and 389 (24.4%) adults. The samples were subjected to MAC ELISA and IgG ELISA.

**Results:**

Of the 968 patients, 686 (43.0%) were positive, of which 579 (84.0%) were in the paediatric age group (<14 yr) and 107 (15.5%) were adults. The IgM positivity being 356 (36.7%) in males and 330 (52.8%) in females. Of the 686 positives, 113 (16.47%) were positive for both IgM and IgG denoting secondary infection. There was a noticeable increased occurrence during the cooler months and during the monsoon and post-monsoon months.

**Interpretation & conclusions:**

The dengue IgM seropositivity among the suspected cases indicates active dengue virus activity. Increase in the probable secondary infections especially in a country like ours where multiple serotypes are prevalent raises concern over probable increase in the incidence of the more serious DHF/DSS. Studies need to be done to identify circulating serotypes of dengue virus to design preventive strategies.

Outbreaks of illness clinically resembling dengue fever (DF) have been there ever since 1779 in Java, Indonesia. Similar epidemics of dengue like illness occurred at 10-30 yr interval. Now the spread has been accelerated by the advent of frequent air travel^1^. Majority of the infections are asymptomatic and the clinical manifestations occur in two forms- classical dengue fever and dengue haemorrhagic fever/dengue shock syndrome (DHF/DSS). DHF/DSS are uncommon in individuals above 15 years and are more common in secondary infections^2^–^4^. Several virus- and host-specific factors have been suggested to correlate with severe disease outcome, which are mostly associated with secondary infections^3^,^4^.

Dengue infection has been known to be endemic in India for over two centuries, as a benign and self-limiting disease. One of the largest outbreaks in north India occurred in Delhi and adjoining areas in the 1996 which was mainly due to dengue-2 virus^5^. Thereafter, in 2003, another outbreak occurred in Delhi and all four dengue virus serotypes were found to be co-circulating^6^–^8^. However, dengue-3 was reported to predominate in certain parts of North India in 2003^9^. In the following years (2004 and 2005), though outbreaks did not occur but a high number of cases of suspected dengue infection were reported during rainy season. The seasonality of transmission of dengue showed increased activity during monsoon and post monsoon. These findings indicate that during epidemic as well as non-epidemic years, dengue infections are mostly seen in monsoon and post-monsoon season.

Dengue has been rampant in parts of Tamil Nadu in the past two decades. The prevalence of dengue vector and silent circulation of dengue viruses have been detected in rural and urban Tamil Nadu, which is ever increasing^10^.

In this context, a retrospective analysis of data was done on the samples received for dengue testing at King Institute of Preventive Medicine, Guindy, Chennai, Tamil Nadu, during three years from 2006 to 2008. The serum samples from clinically suspected cases referred to Virology laboratory for IgM and IgG testing were included in the study. The results were analysed to investigate whether there is an overall increase in the dengue prevalence over the three years period.

## Material & Methods

The study was performed at the King Institute of Preventive Medicine, Department of Virology, Guindy. Samples were received from Government General Hospital, Institute of Child Health, other Government hospitals and private institutions all over Chennai. Since these are tertiary care centres, the samples received were inclusive of referred patients from the various districts in Tamil Nadu. Both adult and paediatric cases were included in this study.

The patients were referred to as suspected DF cases, based on standard diagnostic criteria^1^. Most of the samples were collected during 5 to 10 days of illness. Approximately 2-5 ml of blood was received, serum separated and subjected to ELISA. MAC ELISA was performed using kit from Panbio, IgM Capture ELISA (Australia) and IgG ELISA was also performed using kits from EUROIMMUNO AG DEUTSCHLAND, SEELEAMP 31. A few representative cases during the year 2006, 2007 and 2008 were subjected to Rapid test, PAN BIO DUO CASETTE (Australia) in which IgG and IgM can both be detected.

*Statistical analysis*: The data presented were analyzed using Chi-square test for proportion and the Chi-square test for linear trend using the Graphpad prism 5.02 programmes.

## Results

During the study period, the total number of samples screened was 1593 of which 686 (43.0%) were positive for IgM antibodies (Table I). There was an increase in the percentage positivity in 2008 when compared to 2006 (*P*<0.05).

**Table I:** Year-wise distribution of suspected cases of dengue fever and dengue IgM positive cases over a three year period (2006-2008)

Year	Total no. of suspected dengue cases	Total no. (%) of IgM positives

2006	578	204 (35.2)
2007	517	232 (44.8)
2008	498	250 (50.2)
Total	1593	686 (43.0)*

* *P*<0.05 compared to 2006.

Of the 1593 cases screened (968 males, 625 females), the IgM positivity was 356 (36.7%) in males and 330 (52.8%) in females (Table IIa). The overall increase in the seropositivity among males during the study period was found to be statistically significant (*P*<0.05).

**Table IIa:** Year-wise and gender-wise distribution of suspected cases of dengue fever and dengue IgM positive cases over a three year period (2006-2008)

Year	Male		Female		
	Total no. of suspected dengue cases	No. of positives (%)	Total no. of suspected dengue cases	No. of positives (%)

2006	370	110 (29.7)	208	94 (45.1)
2007	300	120 (40.0)	217	112 (51.6)
2008	298	126 (42.2)	200	124 (62.0)
Total	968	356 (36.77)	625	330 (52.8)

**Table IIb:** Year-wise and age-wise (children and adults) distribution of suspected cases of dengue fever and dengue IgM positive cases over a three year period (2006-2008)

Year	Children		Adults	
	Total no. of suspected dengue cases	No. of positives (%)	Total no. of suspected dengue cases	No. of positives (%)

2006	448	182 (40.6)	130	22 (16.9)
2007	388	195 (50.2)	129	37 (28.6)
2008	368	202 (54.8)	130	48 (36.9)
Total	1204	579 (48.08)	389	107 (27.50)

**Table III:** IgM year-wise positivity in paediatric age group

Year	Age(yr)				Total no. of IgM positives
	<1	1-5	6-12	13-18	

2006	48 (26.3)	80 (43.9)	30 (16.4)	24 (13.1)	182
2007	40 (20.5)	80 (41.0)	40 (20.5)	35 (17.9)	195
2008	37 (18.3)	77 (38.1)	50 (24.7)	38 (18.8)	202
Total	125 (21.58)	237 (40.93)	120 (20.75)	97 (16.75)	579

^*^values are no. (%)

Of the 1593 cases screened, 1204 (75.5%) were paediatric cases and 389 (24.4%) were adults. Of the 686 reactive cases, 579 (84.5%) were positive paediatric cases (<14 yr) and 107 (15.5%) were adults (Table IIb). The samples were collected among the age group of 0-89 yr and the mean age was (19 ± 17 yr). The overall increase in the seropositivity among paediatric and adult cases was statistically significant (*P*<0.05). Among the paediatric age group, positivity was significantly high (*P*<0.001) in 1-5 and 6-12 yr age group (Table III).

Of the 686 positives, 113 were positive for both IgM and IgG denoting secondary infection. The percentage of samples positive for both IgM and IgG was found to be more in 2007 and 2008. There could be a co-circulation of different strains causing increased secondary infections. IgM positives are inclusive of both isolated IgM positives and those positive for both IgM and IgG. There was an overall increase in the secondary infections during the three years and was found to be statistically significant (*P*<0.0001).

The observed dengue IgM seropositivity month wise is illustrated from 2006-2008. (Fig.). The percentage of IgM positivity was found to be high during the months of September and October in all the three years. The observed dengue IgM seropositivity percentage showed an increase with increase in the monthly rainfall. The IgM seropositivity percentage showed temporal relationship with fall in temperature. There was an increased seropositivity during the cooler months.

**Fig.**
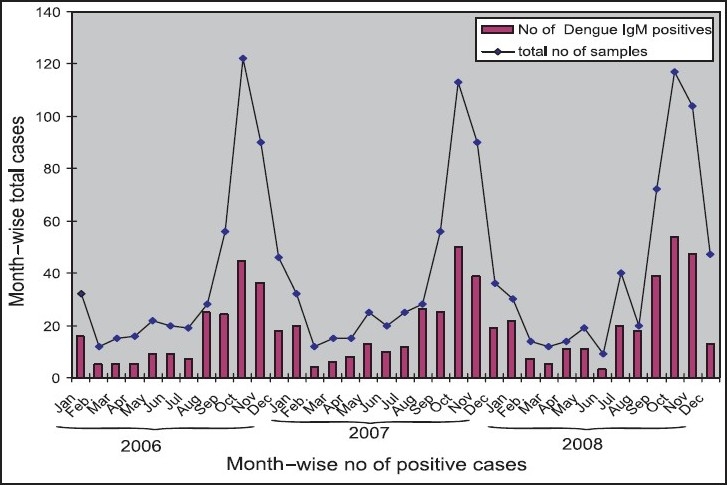
Month-wise distribution of suspected cases of dengue fever and dengue IgM positive cases over a three year period (2006-2008).

The increase in the number of dengue IgM seropositivity among paediatric cases as compared to adults was statistically significant (*P*<0.005).

## Discussion

The first isolation of dengue virus serotypes 1and 4 was reported from India in 1964^11^,^12^, and dengue virus serotype 3 in 1996^13^. Ever since, intermittent reports of dengue and its sequelae have come from various parts of the country. These include reports from Ludhiana^14^, Delhi^8^, Lucknow^13^, Kolkata^15^, Chennai^16^, Mangalore^17^, Assam/Nagaland^18^ and Vellore^19^–^21^. The present study showed a steady increase in the number of referred cases and increase in the percentage of samples positive for dengue IgM during the study period.

The vulnerability of children to dengue infection was re-established in our study, as has been reported earlier^20^.

The transmission of dengue increases in monsoon^22^, as was also observed in our study. This shows that the presence of stagnating water after rainfall favours breeding of the mosquito vector resulting in an increased incidence of dengue. These findings also indicate that preventive measures against dengue infection should probably come into full-swing during the monsoon and post monsoon months.

Molecular studies on the circulating serotypes and their genotypes may be of help in addressing the probabilities of DSS/DHF incidence in future. Involvement of many laboratories in diagnosis of dengue coupled with general awareness among the public and constant vigilance by the health care officials could go a long way in combating dengue.
